# Technical Diagnosis on Elite Female Discus Athletes Based on Grey Relational Analysis

**DOI:** 10.1155/2022/8504369

**Published:** 2022-04-15

**Authors:** Dong Chen, Xuejun Ma, Jiawei Dong

**Affiliations:** China Athletics College, Beijing Sport University, Beijing 100085, China

## Abstract

The personalized training of elite athletes is the key to the breakthrough of Chinese track and field events in the Tokyo Olympic Games. The brilliance of Chinese women's throwing benefits from the closed-loop personalized training system. Training starts from the construction of accurate personalized technology and physical fitness model. This paper introduces the concept of champion model and puts forward a targeted technical training system based on the differentiation research of champion model. This paper mainly studies and analyzes some important technical parameters and achievements of elite female discus athletes aged 2018∼2021 by using the methods of Pearson, partial correlation and grey correlation analysis. We select from several technical parameters with significant differences, choose from several technical parameters that have significant difference, and calculate the correlation parameters and results. The results show that the influence degree of these technical parameters is as follows: torso angle of right foot touching the ground, discus release angle, discus release speed, shoulder and arm passing angle of left foot off the ground, discus moving distance of double support, center of mass moving distance of double support, and time of the second single support stage. This is different from our view that the hand speed is the most important, so the training of elite athletes should be more refined and specialized to promote the improvement of their performance. Through the application of technical diagnosis model in Chen Yang in a period of time, Chen Yang got her best result (65.14 m) in Chongqing Athletics Championship, which verified the success of the technical diagnosis model and application in this study.

## 1. Introduction

Technical training is the key content of discus thrower's training system, and it is the key factor to determine the athletes' performance in competition. Good technical training in competition is the important guarantee to obtain excellent results, and selecting the key part of technical training which contributes more to the results can get twice the result with half the effort. For elite athletes, the impact of injury, especially the serious injury that needs to stop training or even surgery, will lead to the forced suspension of training or competition, hinder the improvement of sports performance, shorten sports life, and even lead to disability in serious cases. Sports injury is an important factor that restricts high-level athletes to achieve better results and even directly leads high-level athletes away from sports venues. Therefore, it is of great practical significance to study the sports injury factors of elite discus athletes.

This paper analyzes Chen Yang's results in five important competitions and 18 throws in four years and tries to find out the important parameters of Chen Yang's technical characteristics which have an important impact on her performance, so as to provide reference for her future preparation for the Olympic Games and the National Games, as well as some theoretical research methods for the majority of trainers. The validity of the technical diagnosis model was verified through the competition.

This paper mainly uses the grey correlation analysis to obtain the correlation degree between the key technologies and achievements of excellent female discus athletes, adopts the corresponding training methods and means in the training, and introduces the special strength problem under the technical response. After special strength training, they participated in the competition and achieved good results, which verified that the technical model construction of this study is effective. The technical parameters of Chen Yang's results in the competition from 2018 to 2021 and the competition results after effective training were studied and analyzed. In traditional analysis methods, when athletes suffer from sports injuries, the rehabilitation process usually focuses on the recovery of the body. This ignores the importance of athletes' thoughts, emotions, and motivation in the process of successful rehabilitation. These experiences focus on the whole process of sports injury, not just the physiological or psychological aspects of sports injury and rehabilitation. The research mainly analyzes the characteristics of major sports injuries of high-level athletes, so as to provide reference for athletes and coaches to avoid or reduce the occurrence of injuries.

## 2. Construction of Women's Discus Champion Model Based on Grey Correlation Analysis

### 2.1. Division of Discus Movement Stages

**Figure 1:**
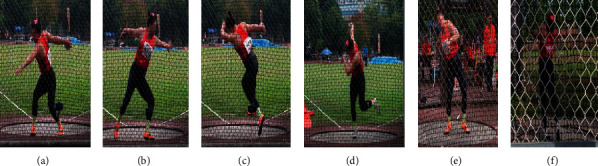
Discus technology time phase and phase division diagram. (a) Maximum preswing, (b) moment of right foot off the ground, (c) moment of left foot off the ground, (d) moment of right foot touching the ground, (e) moment of left foot touching the ground, and (f) moment of release (double support stage ⟶← first single support stage ⟶← aerial stage ⟶← second single support stage ⟶← force stage).

In order to facilitate the relevant technical analysis of this study, the author divides the complete discus throwing technology with reference to scientific researchers. This paper divides it into six time periods and five stages (as shown in Figure 1): maximum backward swing of throwing arm, right foot off the ground, left foot off the ground, right foot touching the ground, left foot touching the ground, release moment, double support stage, first single support stage, airborne stage, second single support stage, and final force stage [1].

### 2.2. Analysis of Screening Results of Technical Indexes Related to Discus Results

**Figure 2:**
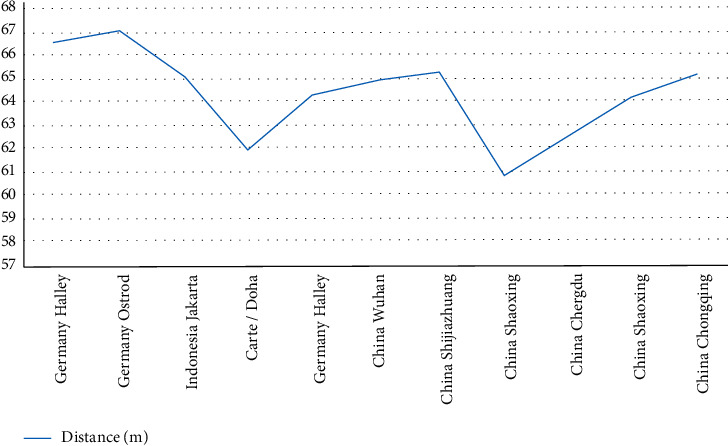
Important competitions and reference results from 2018 to 2021.

The Pearson partial correlation method is used [2]. For each key point in different stages, displacement of discus in different stages is different with the difference of speed, angle, and shooting height [3], the key points in different stages of the transcendence of shoulder, hip angle, each key point angle, the key to the trunk angle, the arm angle for a total of 10 key indicators and performance analysis of the correlation, the following Table 1, for evaluation index selection index R, P. In general, the closer the absolute value of *R* is to 1, the greater the correlation with performance is [4]. There is a significant difference between *P* value <0.05 and performance. The important competitions and reference results from 2018 to 2021 are shown in Figure 2.

From Table 1, we can see that the indicators that are correlated with the results are discus release speed (*R* = 0.609, *P*=0.007), left foot off the ground shoulder and arm overtaking angle (*R* = −0.482, *P*=0.043), and right foot off the ground trunk angle (*R* = −0.466, *P*=0.041), and second support time (*R* = 0.603, *P*=0.008), double support stage displacement (*R* = 0.492, *P*=0.038), double support stage centroid displacement (*R* = 0.575, *P*=0.013), and shot angle (*R* = 0.531, *P*=0.023) are correlated with performance. Another parameter that coaches pay attention to is the shoulder hip torsion angle [5]. However, the comparative study of Chen Yang's achievements over the years did not find an obvious correlation with her achievements, which may also have a certain relationship with the technology of studying Chen Yang's individual [6].

### 2.3. Grey Correlation Analysis of Chen Yang's Technical Indicators and Achievements

Grey correlation analysis is used in this paper [7]. This paper calculates the correlation degree between technical indicators and results of Chen Yang, China's elite female discus athlete in the four years competition, and analyzes the correlation degree between Chen Yang's technical indicators and results [8]. The specific research and analysis steps are as follows.

### 2.4. Select the Reference Value of Effect Measure in Association Analysis

Seven important indexes with significant differences in Chen Yang's discus technical achievements in the past four years were selected for correlation analysis [9], the results of the competition as a reference sequence (X0), and the selected seven technical indicators as a comparison sequence, according to the analysis principle of grey correlation analysis to select the reference value in each sequence. For the discus competition result (X0), the larger the selected data, the better. Therefore, *X*0 = 65.42 m. Among discus technical indexes, the larger the index is, the greater the contribution to the result will be within a certain range [10]. Therefore, in the comparison sequence X1–X7, the best value should be selected for each sequence. The best value of each comparison sequence is as follows: *X*1 = 25.82 m/s, *X*2 = 39.74°, *X*3 = 24.76°, *X*4 = 42.87°, *X*5 = 0.14 s, X6/*m* = 2.04, and *X*7 = 0.24 m. The selected original data are shown in Table 2.

### 2.5. Conduct Dimensionless Processing on Selected Data

Because of the different physical meanings of the selected technical indicators, the dimensions of the data may not be the same [11], which is not conducive to comparison and may cause certain errors in the results. Therefore, it is necessary to carry out dimensionless mean processing for the original data. According to the reference values of the selected data [12, 13], they are substituted into the following formula for dimensionless mean processing:

(1) $$\begin{array}{c} Y_{i}\left(k\right)=\frac{X_{i}\;\left(k\right)}{X_{i}\left(k\right)\max}, \end{array}$$

where *Y(k)* represents the dimensionless value of the *i*th comparison sequence and Xi (*k*) max represents the optimal value in the *i*th sequence data.

### 2.6. Calculation of Correlation Coefficient between Discus Technology and Performance

The degree of correlation is actually the degree of difference between geometric shapes in space, so the difference between curves can be used as a measure of the degree of correlation, that is, the comparison sequence X1, X2, and X3 with seven technical indicators for the reference sequence discus score X0 … X7; the correlation coefficient between the comparison sequence of each technical index and the reference sequence at each time (each point in the curve) can be calculated by the following equation, where *ρ* is the resolution coefficient, generally between 0 and 1, usually with the value of 0.5 [14].

△ is the second-order minimum difference, denoted as △min, and is the two-order maximum difference, denoted as △max.

The absolute difference between every point on the Xi curve of each comparison sequence and every point on the X0 curve of reference sequence is denoted as △0i(k) [15].

The dimensionless reference sequence Y0 is taken as the reference standard, and the correlation coefficient () between the dimensionless comparison sequence Y1–Y7 and the reference sequence Y0 can be calculated, which can be simplified into the following formula, according to which the correlation coefficient 1–7 between each technical indicator and achievement can be calculated, respectively, *δδ*(*xi*)*δ* (Table 3).

(2) $$\begin{array}{c} \delta_{0i}\frac{△\min+\rho △\left(\max\right)}{△0ik+\rho △\left(\max\right)}. \end{array}$$

### 2.7. Calculation of Correlation Degree between Chen Yang's Technology and Grades

The literature compares the connection degree of each torque value in the reference sequence (technical index) [16], because information is scattered integrity is not easy to compare, so every moment of the correlation coefficient of concentration of a numeric value [17, 18], namely, the average, as a result the number of degree of associated with various technical indicators, said. The correlation degree can be calculated by the following formula (R1–R7):

(3) $$\begin{array}{c} {\text{r}}_{\text{i}}=\frac{1}{N}\sum_{K=1}^{N}\delta_{i}\left(k\right). \end{array}$$

**Figure 3:**
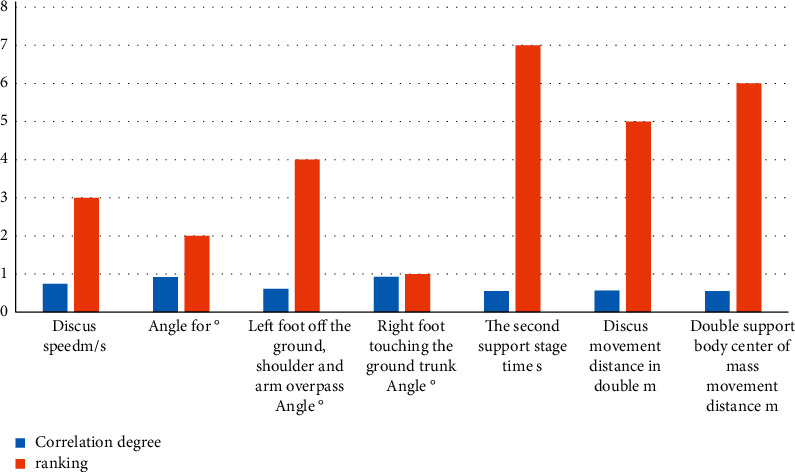
Correlation degree between each technical index and achievement.

The correlation between technical indicators and performance is shown in Figure 3. The seven evaluation items include discus release speed (m/s), discus release angle, left foot off the ground shoulder and arm crossing angle, right foot touching the ground torso angle, second single support stage time *s*, discus movement distance *m* in double support stage, and double support body centroid movement distance *m* and result *m* (correlation) [19]. The research also provides an analysis reference based on correlation degree. The discrimination coefficient used in grey correlation analysis is 0.5 [20]. Calculate the correlation value in combination with the correlation coefficient calculation formula, and then judge the correlation between Chen Yang's important indicators and 21 technical achievements according to the correlation value [21].

The correlation degree value is between 0 and 1, and the larger the value is, the stronger the correlation is with the “reference value” (discus result), that is, the higher the evaluation is. As can be seen from the above table, the correlation degree between discus technical parameters and results in Chen Yang's competition data in the past four years is R4 R2 R1 R3 R6 R7 R5. That is, the influence of various technical indicators on discus results from large to small is as follows: torso angle of right foot touching the ground, discus release angle, discus release speed, shoulder and arm passing angle of left foot off the ground, discus moving distance in double support stage, and moving distance of double support body center of mass and time in the second single support stage [22].

From the above sequence and table, we can intuitively see the whole empty technical action after the active throwing discus falls to the ground. The trunk angle of the right foot touching the ground is the shooting angle. The discus throwing performance has the greatest correlation with the technology emphasized by coaches in training practice. That is, after landing, a good empty torso angle is kept, the left arm back is thrown, and the body is kept moving above the machine as much as possible [23].

Chen Yang, the individual in this study, is an excellent discus thrower in China and the best representative of women's discus thrower in China at the present stage. The improvement of her performance is no longer a simple strength training and the training of the whole throwing technique, but we should focus on a certain point in the training of throwing technique [24]. For example, in this study, the landing torso angle of the right foot and the hand angle are arranged according to the importance of techniques.

## 3. Analysis and Discussion of Calculation Results

After obtaining Chen Yang's technical diagnosis model, the coaches and the team's scientific research personnel adopted targeted training methods for Chen Yang's technical weaknesses. The targeted training of physical strength and skills was adopted from the first half of 2021 [25]. In June 2021, Chen Yang participated in the National Track and Field Championship & Olympic Trials and the National Track and Field Championship & National Games qualification and achieved good results, especially in the Chongqing Track and Field Championship, which created Chen Yang's personal best of the year (65.14 m).The specific training of physical strength and skills is mainly based on several kinematic parameters that are highly correlated with the results, such as torso angle when the right foot touches the ground, discus release angle, discus release speed, overshooting of shoulder arm when the left foot leaves the ground, discus displacement and centroid displacement in the double support stage, and the second single support time.

In recent years, Chen Yang's several indicators with high correlation with her performance reflected in her physical strength and technique can be summarized as the following shortcomings: the angle of her right foot touching the ground and the time of the second support belong to the second support stage. Discus release angle and discus release speed are both motion parameters in the final effort. The double support phase displacement and centroid displacement reflect the discus motion parameters in the starting stage. The initial stage of discus is more important than the athletes' mastery of technology [26], and the final stage of exertion is the joint effect of the athletes' body quality and technology. To solve these problems, coaches and researchers mainly adopt the following training modes and means, as shown in Table 4.

For the torso angle of right foot touching the ground in the second single support stage, Chen Yang mainly adopted techniques of strengthening the body's lower limb strength, ankle strength, and right hand holding cake. Chen Yang mainly adopts the training mode of strength + technique + speed. The training mainly focuses on strengthening the release timing, overall burst, and power speed. In the initial stage of discus displacement and centroid displacement, strength + technique was mainly used to strengthen Chen Yang's ability to make the maximum backswing on the cake and strengthen shoulder joint flexibility and upper limb strength.

Coaches and team after the construction of diagnosis model for active technology focused on the technical parameters of active training, and after a period of targeted and strengthened training, he participated in the national track and Field Championships and Olympic qualifiers, as well as the national track and Field Championships and National Games qualifiers. They all achieved good results, especially in the championship, and they actively threw 65.14 meters [27]. She created the best result of this season, which is closely related to the technical diagnosis model built by Chen Yang through her own years of competition. The targeted training of her technical parameters is the boosting agent to promote her performance progress. The performance and kinematic parameters of Chen Yang before and after applying the technical diagnosis model are shown in Table 5.

From the technical diagnosis model of the active years' grades and scores rank correlation, through targeted training, active in track and field championships in Chongqing right foot touches the ground of the torso angle has great growth, trunk angle increase represents the active after landing by completing empty body torso angle with the ground vertical angle increases. From the point of view of discus technology, it means that Chen Yang's flying and landing technology is relatively excellent in this competition, and her body can maintain a good movement beyond the apparatus. Good overstepping apparatus movement is the guarantee of forming a good final power movement. From the perspective of other kinematic parameters, some improvements were made in this competition. However, because of the short time of targeted training, the benefits were not so obvious. Therefore, it is necessary to continue to strengthen targeted training, in order to achieve great improvement in the following Olympic Games and National Games, so as to improve sports performance.

## 4. Discussion on Psychological Characteristics of Athletes

Through the investigation, it is found that the psychological factors of athletes' sports injury mainly include depression in training or competition, excitement in training or competition, inattention, poor awareness of self-protection, and so on. The low excitement in training or competition is mainly due to athletes' physical discomfort, satisfaction with training or competition arrangements, or other reasons, resulting in athletes' unwillingness to participate in training or competition. In other words, athletes' training and competition come from psychological resistance. In actual training or competition, athletes will continue to train and compete with their scalp in order not to be criticized.

When athletes are nervous, the coach can take language guidance and muscle massage as a supplement. Athletes can also use deep breathing, meditation, and other methods to regulate inner tension. However, some inexperienced athletes may not know when they are nervous. With the fast development of computer techniques, in the future, facial expressions of athletes can be accurately tracked and recognized via computer techniques to confirm if they are nervous [28–30]. If nervous, their coaches can take actions to help the athletes.

## 5. Conclusion

The correlation degree of the relevant technical data and performance data of Chen Yang's four-year competition is as follows: Discus performance is related to seven technical indicators, right foot landing torso angle. It is the most important point that the correlation between angle and result is the largest, even faster than we usually think. Moreover, it may also have a certain relationship with the individual differences of initiative, but from the calculation results, it meets the training requirements of coaches. However, the training methods and means may need to be improved to strengthen the technical trace effect of Chen Yang's training and promote the improvement of her sports performance.

After a period of application of the technical diagnosis model, Chen Yang's technology has been improved in the subsequent competitions and her performance has also been correspondingly improved. She created the best result of this year in the Chongqing Track and Field Championship. Therefore, the establishment and application of Chen Yang's multiyear technical diagnosis model are successful.

With the progress of science and technology and the development of interdisciplinary integration, scientific training, targeted training, and personalized training of elite athletes have increasingly become the development trend of competitive sports; the traditional training mode aims to enable athletes to obtain competitive ability through repeated repetition, but the old track of special technology is difficult to change through repetition, especially changing neural pathway and muscle memory. The target positioning of special technical and physical training is not clear, which also seriously restricts the training benefits. The grey correlation analysis based on the athlete champion model can effectively improve the athlete's short board positioning and target positioning and make the direction of technical correction and physical improvement more clear.

After the empty stage, right foot landing trunk angle and the angle of technology in addition to the basic training method can also be combined with muscle power, electrical muscle power to its timing sequence, and the degree of brain cortex and monitoring in order to better promote the improvement of active technology and thus the improvement of its performance of power, for the Olympic Games and National Games to achieve better results to provide a certain practical significance.

However, this paper does not pay attention to the analysis and training of athletes' psychological state in the research. In the direction of psychological science power, especially for the state analysis training of elite athletes, when their sports ability or performance develops to a personal bottleneck, scientific and technological power often has limited benefits. In the future research, it is also necessary to study the physical fitness analysis from the psychological perspective of athletes.

## Figures and Tables

**Table 1:** Correlation analysis between each key point or stage and achievement.

	After put the biggest	Right foot off the ground moment (double support)	Left foot off the ground (first shot support)	Right foot touching the ground (airborne)	Moment left foot touches the ground (second support)	Moment of release (force stage)
Discus speed		*R* = 0.238, *P*=0.341	*R* = 0.249, *P*=0.318	*R* = 0.409, *P*=0.092	*R* = 0.075, *P*=0.769	*R* = 0.609, *P*=0.007

Angle of shoulder and arm overtaking angle	*R* = 0.020, *P*=0.938	*R* = 0.373, *P*=0.128	*R* = 0.482, *P*=0.043	*R* = 0.183, *P*=0.468	*R* = 0.310, *P*=0.210	*R* = 0.335, *P*=0.174

Shoulder hip angle of transcendence	*R* = 0.366, *P*=0.135	*R* = 0.101, *P*=0.690	*R* = 0.259, *P*=0.300	*R* = 0.225, *P*=0.368	*R* = 0.336, *P*=0.173	*R* = 0.102, *P*=0.687

The trunk angle	*R* = 0.344, *P*=0.162	*R* = 0.466, *P*=0.041	*R* = 0.89, *P*=0.724	*R* = 0.437, *P*=0.063	*R* = 0.217, *P*=0.387	*R* = 0.456 *P*=0.057

Arm angle	*R* = 0.265, *P*=0.287	*R* = 0.061, *P*=0.808	*R* = 0.398, *P*=0.102	*R* = 0.405, *P*=0.095	*R* = 0.155, *P*=0.540	*R* = 0.377, *P*=0.123

When		*R* = 0.332, *P*=0.178	*R* = 0.540, *P*=0.021	*R* = 0.477, *P*=0.045	*R* = 0.603, *P*=0.008	*R* = 0.099 *P*=0.698

Discus displacement		*R* = 0.492, *P*=0.038	*R* = 0.153, *P*=0.545	*R* = 0.464, *P*=0.052	*R* = 0.449, *P*=0.062	*R* = 0.048, *P*=0.849

Centroid displacement		*R* = 0.575, *P*=0.013	*R* = 0.033, *P*=0.897	*R* = 0.549, *P*=0.018	*R* = 0.458, *P*=0.056	*R* = 0.185 *P*=0.463

To highly						*R* = 0.263, *P*=0.292

Angle for						*R* = 0.531, *P*=0.023

The absolute value of *R* is close to 1, indicating a greater correlation with performance; *P* < 0.05 was significantly different from grade.

**Table 2:** Reference and comparison of sequence raw data.

Serial number	1	2	3	4	5	6	7	8	9	10
The competition result is X0 (m)	62.6	64.26	64.46	65.42	63.4	64.55	62.22	62.44	62.66	61.88
Discus hand speed X1 (m/s)	24.36	24.71	25.02	24.65	24.58	25.82	23.42	24.47	24.08	24.82
Discus release angle X2 (°)	39.22	38.88	38.11	37.04	38.99	39.74	38.39	35.92	38.32	34.57
Left foot off the ground, shoulder and arm overpass angle X3 (°)	0.24	2.21	4.05	4.77	16.03	16.13	24.76	4.72	4.19	8.39
Right foot touching the ground trunk angle X4 (°)	42.06	42.87	41.4	38.53	31.71	33.32	39.16	37.84	37.88	39.86
The second support stage time is X5 (s)	0.18	0.2	0.16	0.16	0.17	0.18	0.17	0.15	0.16	0.14
Discus movement distance X6/*m* in double support stage	2.04	1.88	1.87	1.92	1.87	1.86	1.32	1.24	1.44	1.45
Double support body center of mass movement distance X7 (m)	0.32	0.31	0.35	0.32	0.26	0.25	0.25	0.24	0.26	0.28

**Table 3:** Correlation coefficients between reference sequence and comparison sequence.

Serial number	1	2	3	4	5	6	7	8	9	10
Y0	0.96	0.98	0.99	1.00	0.97	0.99	0.95	0.95	0.96	0.95
Y1	0.94	0.96	0.97	0.95	0.95	1.00	0.91	0.95	0.93	0.96
The delta 1	0.99	0.97	0.98	0.95	0.98	0.99	0.95	0.99	0.97	0.98
Y2	0.99	0.98	0.96	0.93	0.98	1.00	0.97	0.90	0.96	0.87
The delta 2	0.97	1.00	0.97	0.93	0.99	0.99	0.98	0.95	0.99	0.92
Y3	0.01	0.09	0.16	0.19	0.65	0.65	1.00	0.19	0.17	0.34
The delta 3	0.01	0.09	0.17	0.19	0.67	0.66	0.95	0.20	0.18	0.36
Y4	0.98	1.00	0.97	0.90	0.74	0.78	0.91	0.88	0.88	0.93
The delta 4	0.98	0.98	0.98	0.90	0.76	0.79	0.96	0.92	0.92	0.98
Y5	0.90	1.00	0.80	0.80	0.85	0.90	0.85	0.75	0.80	0.70
The delta 5	0.94	0.98	0.81	0.80	0.88	0.91	0.89	0.79	0.84	0.74
Y6	1.00	0.92	0.92	0.94	0.92	0.91	0.65	0.61	0.71	0.71
The delta 6	0.96	0.94	0.93	0.94	0.95	0.92	0.68	0.64	0.74	0.75
Y7	0.91	0.89	1.00	0.91	0.74	0.71	0.71	0.69	0.74	0.80
The delta 7	0.96	0.90	0.99	0.91	0.77	0.72	0.75	0.72	0.78	0.85

**Table 4:** A brief list of targeted exercises that Chen Yang adopted according to the technical diagnosis model.

Motion parameters	Practice mode	Practice means	Exercise load
The initial stage	Strength + flexibility + technique	Bench press barbell exercises (2) Practice with the hose in place (3) Rotate and swing the hose (4) KEISER-half squat with arms pulled back	In the

Second single support stage	Strength + technology	(1) KEISER-standing and sitting straight arm rotation exercise (2) KEISER-stand and sit heel raises (3) KEISER-hip flexor and hip flexor exercises (4) KEISEER-stand pose spin pull exercise (5) Combination practice of big hurdle	Small

Final force stage	Strength + skill + speed	(1) Sitting posture holding a small piece of chest stretching + pushing exercise (2) Shot put practice before and after (3) Bench barbell hip press exercises (4) Skip boxes (5) Tall cup squats (6) Barbell high rolls and squats 7.30 meters running practice	Big

**Table 5:** Chen Yang's performance and kinematic parameters before and after applying the technical diagnosis model.

Serial number	Competition result (m)	Discus hand speed (m/s)	Discus release angle (°)	Left foot off the ground, shoulder and arm exceed angle	Right foot touching the floor torso angle (°)	Second single support stage time (s)	Discus movement distance *m* in double support stage	Center of mass movement distance *m* of double supporting body
1	62.6	24.36	39.22	0.24	42.06	0.18	2.04	0.32
2	64.26	24.71	38.88	2.21	42.87	0.2	1.88	0.31
3	64.46	25.02	38.11	4.05	41.4	0.16	1.87	0.35
4	65.42	24.65	37.04	4.77	38.53	0.16	1.92	0.32
5	63.4	24.58	38.99	16.03	31.71	0.17	1.87	0.26
6	64.55	25.82	39.74	16.13	33.32	0.18	1.86	0.25
7	62.22	23.42	38.39	24.76	39.16	0.17	1.32	0.25
8	62.44	24.47	35.92	4.72	37.84	0.15	1.24	0.24
9	62.66	24.08	38.32	4.19	37.88	0.16	1.44	0.26
10	61.88	24.82	34.57	8.39	39.86	0.14	1.45	0.28
X	63.39	24.59	37.92	8.55	38.46	0.17	1.69	0.28
X + SD	63.89 ± 1.14	24.59 ± 0.59	37.92 ± 1.53	8.55 ± 7.44	38.46 ± 3.41	0.17 ± 0.16	1.69 ± 0.28	0.28 ± 0.036
Shaoxing	63.31	24.36	37.45	11.83	27.3	0.17	1.03	0.28
Chongqing	65.14	24.23	37.32	49.75	43.67	0.18	1.71	0.29

## Data Availability

The data used to support the findings of this study are available from the corresponding author upon request.
